# Effects of exercise programs on kyphosis and lordosis angle: A systematic review and meta-analysis

**DOI:** 10.1371/journal.pone.0216180

**Published:** 2019-04-29

**Authors:** Noelia González-Gálvez, Gemma M. Gea-García, Pablo J. Marcos-Pardo

**Affiliations:** Faculty of Sport, Universidad Católica de Murcia, Murcia, Spain; University of Mississippi Medical Center, UNITED STATES

## Abstract

Many authors are interested in the effects that a specific exercise program could have on sagittal spinal curvatures. The purpose of this study was to determine the effects of different exercise programs on thoracic kyphosis and lumbar lordotic angle. This meta-analysis adhered to the PRISMA guideline and it was registered at PROSPERO. Five electronic databases (Pub Med, Cochrane, WOS, PEDro and EBSCO) were searched up to 31 July 2018. Eligible studies were randomized controlled trials that applied an exercise intervention and measured a kyphosis and/or lordotic angle. Study quality was performance by PEDro score. Risk of bias was assessed using the SIGN 50 checklist for randomized controlled trials. External validity was assessed using the EVAT. Ten randomized controlled trials were included for systematic review and meta-analysis. Meta-analysis with a random effect model was performed to infer the pooled estimated standardized mean difference. All studies were RCTs and they involved a total of 284 cases and 255 controls. Seven studies measured kyphosis angle. A large significant effect of the exercise on kyphosis was identified (SMD = -1.400 (95% CI-2.150 a -0.660), p = 0.000). Four studies assessed lordotic angle and moderate but not significant improvement was shown (SMD = -0.530 (95% CI-1.760 a -0.700), p = 0.401). The results suggest that exercise programs may have a positive effect on thoracic kyphosis angle, but no clear effect on lordotic angle. This systematic review suggests that strengthening rather than stretching could be more relevant for kyphosis and both qualities are important for lordosis. It is necessary to conduct more randomized controlled trials to assess the effects of strengthening and/or stretching program on kyphosis and lordotic angle and to establish the type of the exercise that is better for maintaining the sagittal disposition within normal ranges.

## Introduction

Spinal misalignments are associated different pathologies of the spine such as spondylolisthesis, disc hernias, and certain lesions of acute and chronic characteristics [1], increased intradiscal pressure [2], viscoelastic deformation [3] and back pain [4–6]. Sagittal disposition outside the ranges of normality result in decreased functional capacity and perceived quality of life [7]. In addition, spinal misalignment is associated with a slow gait, poor balance and higher risk of falls [8].

On the other hand, the practice of a systematic and continuous exercise can influence sagittal spinal curvature. Many studies have shown specific spinal adaptations in several athletes such as dancers [9, 10], rhythmic gymnasts [11], users of fitness rooms [12], skiers [13], footballers [14], canoeists [15, 16], tennis players [17], handball players [18], volleyball players [19] and fighters [20]. Repetitive movements of the spine can influence its arrangement through repeated mechanical loads [21]. In addition, hyperkyphosis has been association with low values of hamstring flexibility [22–24] and a lack of abdominal and paravertebral strengthening [24–26]; and hyperlordosis is related with a shortening of psoas iliac (flexor of hip) [24, 27, 28], lack of abdominal and paravertebral strengthening [24, 27] and shortened hamstring muscle [29]. As such, many authors have been interested in the effects that a specific exercise program could have on spinal sagittal curvatures [30–39]. However, to our knowledge, there are no meta-analyses and systematic review of this topic to establish a clear view of the effect of exercise on sagittal curvatures. Therefore, the aim of this systematic review and meta-analysis was to determine the effect of the exercise on thoracic kyphosis and lumbar lordotic angle.

## Materials and methods

### Study design

The studies included in the present meta-analysis and systematic review of the literature examine the effect of exercise on kyphosis thoracic and lumbar lordotic angle. Eligible studies were randomized controlled trials (RCTs). The search strategy, inclusion criteria and additional information were registered in advance on the international prospective registry of systematic review PROSPERO (number CRD42018112718). This meta-analysis adhered to the Preferred Reporting Items for Systematic Reviews and Meta-Analyses (PRISMA) guideline [40, 41].

### Inclusion criteria

The inclusion criteria were: (a) RCTs; (b) exercise intervention with strengthening, stretching, endurance or/and resistance exercise; (c) measurement of thoracic kyphosis or lumbar lordotic angle in grades pre- and post-test, (d) 100% supervised intervention program, and (e) written in English, Spanish or Portuguese language.

The exclusion criterial were: (a) intervention program based on rehabilitation treatment, breathing exercise, mobilization by physical therapist and/or manipulation; and (b) short communication, note, letter, review article or brief report.

### Search strategies

Five electronic databases were searched up to 31 July 2018: PubMed, Cochrane, WOS, PEDro and EBSCO (MEDLINE, CINAHL Complete, SportDiscus with full text, Academic Search Complete, Nursing/Academic Edition, PsycINFO, Family & Society Studies Worldwide, Vocational Studies Complete, Education Source, Psychology and Behavioural Sciences Collection, Health Business Elite, Women´s Studies International, ERIC, Newspaper Source Plus, Dentistry & Oral Science Sources, Food Science Source, Environment Complete, Professional Development Collection, SocINDEX with full text, Regional Business News).

The following search terms and MeSH terms were used: lordosis, kyphosis, spinal curv* or sagittal spinal; combined with the connector AND with other words: exercise, physical activity, program or training. S1 Table shows the complete database search strategy.

#### Data collection and synthesis

Two reviewers (NGG and PJMP) independently used the search terms to screen the literature in the selected databases. They independently screened the titles and abstracts of the search results and reviewed the full text selected for inclusion in the meta-analysis. If there were discrepancies about inclusion, a third reviewer moderated it (GMGG). Cohen´s Kappa was calculated to determinate the inter-rater reliability for the two authors (Kappa = 0.875) and found a strong level of agreement [42].

### Data extraction and risk of bias assessment

Table 1 shows the data extraction of each study. Study quality was performance by the Physiotherapy Evidence Database (PEDro) score. The PEDro score shows strong validity and inter-rater reliability for the evaluation of RCTs [43, 44]. Risk of bias was assessed using the Scottish Intercollegiate Guidelines Network (SIGN 50) checklist for RCTs [45]. External validity was assessed using the External Validity Assessment Tool (EVAT), which measures the generalizability of research to other individuals (external validity) and other settings (model validity) outside the confines of a study [46]. Funnel plots were created and risk of bias was assessed by Egger bias statistics [47] and Rosenthal’s fail-safe N [48].

**Table 1:** Data extraction of each included study.

Author	EG = analy/ recruit; CG = analy/ recruit	Year range; mean±DS	Main inclusion criteria	Main exclusion criterial	Instrument to measure angle (kyphosis/ lordosis)	Programme CG	Programme EG	Time, frequency, duration
Kamali et al 2016 [32]	EG(exercise) = 16/23 CG(manual therapy) = 23/23	18–30 years EG = 23.1±2.3 CG = 23.6±2.9	18–30 years old women with kyphosis angle over 45°	Scoliosis, any spinal disorder history, cancer	Six-camera motion analysis system (kyphosis)	Manual therapy: massage, mobilization, muscle energy and myofascial release	Stretching and strengthening of back muscle	25 min, 3d/wk, 5wk
Muyor et al 2012[39]	EG = 27/27 CG = 31/31	38–50 years 44.23±8.87	35–50 years old women, working (≥8 hours/day) in standing position	Any hamstring or spine disorder or pain over the past six months	Spinal Mouse system (kyphosis / lordosis)	Nothing	Hamstring stretching, 3 unilateral exercise x 20 sec	30 min 3d/wk, 12wk
Kwang-Jun et al 2018[38]	EG1(exercise) = 10/10 EG2(sling) = 10/10 CG = 9/9	EG1 = 43.1±3.7 EG2 = 43.6±4.5 CG = 41.3±3.8	30–40 years old woman with back pain		Radiograph. ViewRex PACS system (lordosis)	Nothing	8 strengthening exercise. 4 exercise equal to EG1 and EG2. EG1 performance the others 4 exercise at mat; and EG2 performance it by slings. 3 set x 10 movement/set	60 min, 3d/wk, 12wk
Jang et al 2017[34]	EG = 22/25 CG = 22/25	Over 65 years EG = 74.6±4.6 CG = 76.8±4.9	Over 65 years old woman with kyphosis angle over 40°		Dual inclinometer (kyphosis)	A guide with the exercise program (self-performance at home)	A guide with the exercise program (same like CG) + thoracic correction exercise with Thera band	60 min, 2d/wk, 8wk
Fatemi et al 2015[31]	EG = 20/20; CG = 20/20	15–18 years EG = 16.10±1.06 CG = 16±1.07	15–18 years old woman with hyperlordosis	Any spinal disorder, exercise or physical therapy during the past two months	Flexicurve ruler (lordosis)	Nothing	William´s training (stretch and strength). 1 set x 10 rep to 3 sets x 20 rep	60 min, 3d/wk, 8wk
Junges et al 2017[35]	EG = 22/22 CG = 19/19	45–78 years 59±9	Over 45 years old women with kyphosis angle over 45°	Smokers, obese, some pathology in the spine	Cobb angle. Panoramic radiograph in profile (kyphosis)	Nothing	Pilates (cadillac, reformer, Wunda Chair, Wall unit, spine corrector, ladder barrel, circles fit) y mat	60 min, 2d/wk, 30wk
Katzman et al 2017[36]	EG = 53/57 CG = 48/55	Over 60 years 70±5.7	Over 60 years old with kyphosis angle over 40°		1) Cobb angle. Spine radiographs 2) Debrunner kyphometer (kyphosis)	Nothing	Multimodal: spinal extensor muscle strength, spinal mobility and postural alignment	60 min, 2d/wk, 12wk
Katzman et al 2017[37]	EG = 43/ 51 CG = 45/48	60–88 years 70.6±0.6	Over 60 years old with kyphosis angle over 40°		1) Cobb angle. Spine radiographs 2) Debrunner kyphometer (kyphosis)	A guide of education program + 60 min/ once a month / 6 month	A guide of education program (same like CG) + Multi-modal group-based kyphosis-specific exercise (strengthening, spinal mobility, spinal alignment	60 min, 3d/wk, 6mth
Hosseinifar et al 2017[33]	EG = 16/16 CG = 16/16	EG = 37.90±9.59 CG = 36±8.69	18–50 years old with chronic LBP more than 3 months	Any spine disorder, pregnancy, cardiovascular diseases, physical therapy	Flexicurve ruler (lordosis)	Routine physiotherapy protocol (TENS, 20 min, and HP, 20 min)	Stretching exercises, low impact aerobic exercises and strengthening exercises aimed at all the main muscle groups	30 min, 6d/wk, 2wk
Seidi et al 2014 [30]	EG1(LCEP) = 19/20 EG2(CCEP) = 18/20 CG = 19/20	18–25 years 20.85±1.7	18–25 years old with kyphosis angle over 42°	Any musculoskeletal disorder, exercise or therapeutic exercise, scoliosis, structural kyphosis	Flexicurve ruler (kyphosis)	Nothing	EG1(LCPE) = local corrective exercise program (kendall´s theory); EG2(CCEP) = comprehensive corrective exercise program	2d/wk, 12wk

DS = standard deviation; EG = experimental group; CG = control group; analy = analysed; recruit = recuited; min = minutes; d = days; wk = weeks

Data extraction and quality assessment was performed by two reviewers (NGG and PJMP) independently. Disagreement about the information was resolved by repeating the data extraction or assessment without observing the reviewer’s previously reported information.

### Statistical analysis

Meta-analysis was completed for continuous data by using the change in the mean and standard deviation between pre- to post-test measurement of the kyphosis and lordotic angle. The studies were grouped according to the assessment angle: kyphosis or lordosis. Some studies reported more than two EGs and were treated like other subgroups in the analysis.

Mean (M) and standard deviation (SD) of the change between pre- and post-test measures of the kyphosis and lordotic angle and the sample size of each group were directly extracted from studies. For studies that did not present the necessary data, SD were calculated and imputed when possible using SEs and Cis. The DerSimonian-Laird (Cohen) pooling method was used and heterogeneity was assessed, using the Cochrane Q test (Chi^2^), Higgins I^2^ and significant (p), to determinate the appropriateness of the application of a fixed or random effect model for the pooled analysis [49]. Meta-analysis with a random effect model was performed to infer the pooled estimated standardized mean difference (SMD) [50, 51]. DerSimonian-Laird (Cohen) was interpreted using Cohen´s [52] as small (0 to 0.2), medium (0.3 to 0.7) and large (≥0.8). The significant differences were determined at a level of p<0.05.

## Results

### Characteristics of the included studies

**Fig 1:**
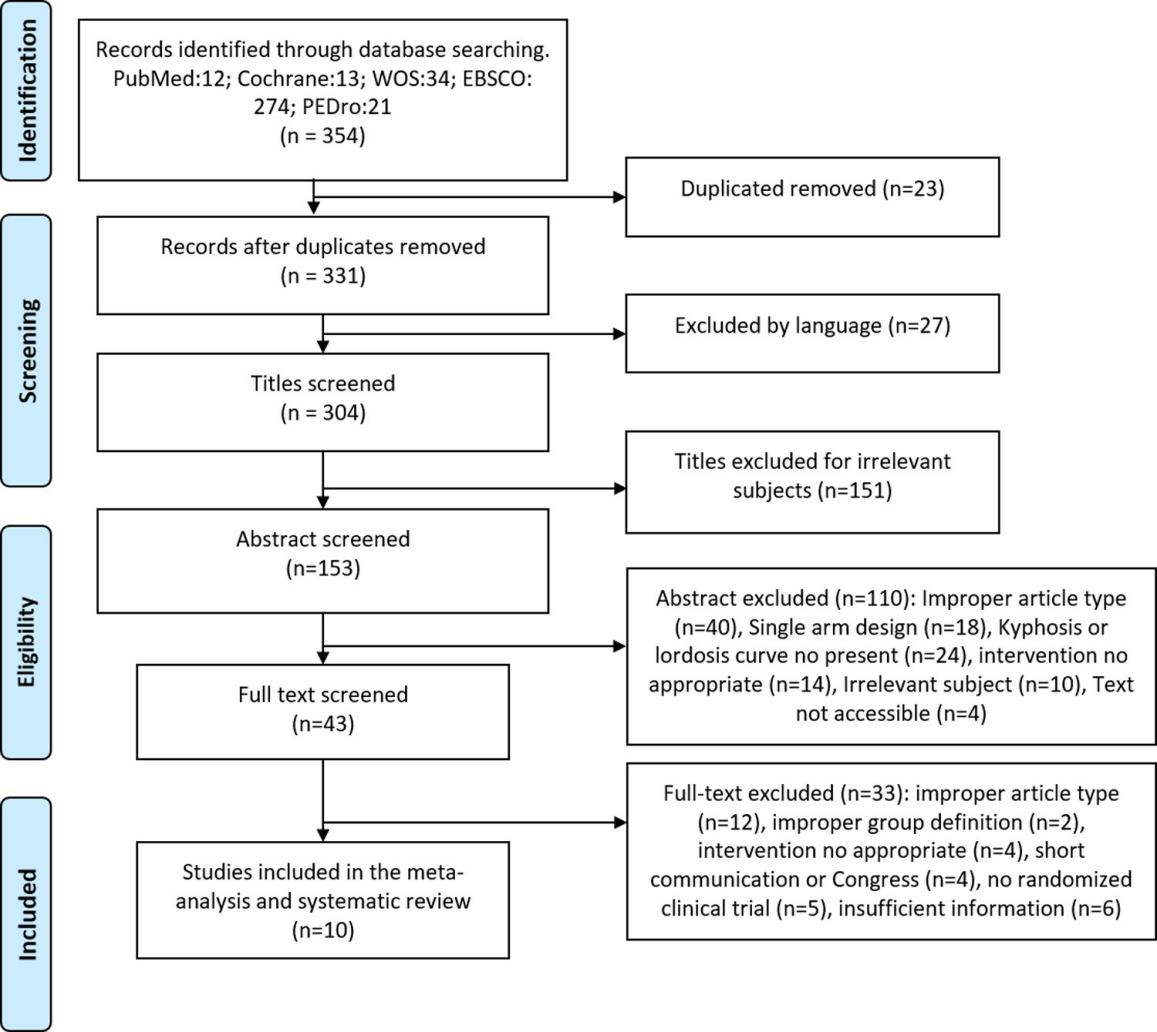
Flow diagram of searched, screened, and included studies.

A total of 354 published were captured and a total of 10 studies were included in this review (Fig 1). The characteristics of the included studies are shown in Table 1. All studies were RCTs and they involved a total of 284 cases and 255 controls.

The mean sample size was 28.4±13.8 (range 16–53) for EG and 25.5±13.1 (range 9–48) for CG. Six studies were carried out with women, [31–33,35,38,39] three studies included women and men, [30,36,37] and one study did not identify the sex [33]. In the studies including women and men, two[36, 37] included a majority of women, 59.4% and 71.1% of women, respectively; in the other study [30], participation was the same between sexes (50% women and 50% men).

For the EG, the mean duration of the exercise programs was 12.5±8.5 weeks (range 2–30), mean frequency was 3±1.2 sessions per week (range 2–6) and 51.88±15.1 minutes per session (range 25–60). All the studies used stretching and/or strengthening exercise in their program as the main objective. Four studies used both stretching and strengthening exercises [32,34,36,37] one study applied just a stretching program [39], one study applied just a strengthening exercise [38], another used stretching and strengthening exercises in addition to low-impact aerobic exercises and strengthening exercises [33], and the rest used a specific method, such as William´s training (stretch and strength) [31], Pilates (Cadillac, reformer, Wunda chair, wall unit, spine corrector, ladder barrel, cycles fit and mat) [35], and a local corrective exercise program (Kendall´s theory) [30]. Eight of the 10 studies did not apply a program in the control group [30,31,34–39]. One study [32] applied manual therapy with massage, mobilization and muscle energy (strength), and another [33] applied a routine physiotherapy protocol (TENS, 20 min, and HP, 20 min).

Six studies evaluated thoracic kyphosis angle [30,32,34–37], three evaluated lumbar lordotic angle [31,33,38], and one evaluated both [39]. Among six studies that evaluated thoracic kyphosis angle, all showed hyperkyphosis as an inclusion criteria [30,32,34–37]. Among three studies that evaluated lumbar lordotic angle, one of them included evidence of lumbar hyperlordosis as inclusion criteria [31]. The other two studies included evidence of low back pain as inclusion criteria [33, 38]. The study that assessed both kyphosis and lordotic angles [39] did not show hyperkyphosis, hyperlordosis or low back pain as an inclusion criteria, they only included those who work at least 8 hours per day in a standing position.

All studies but two used one method, except two that used two methods of measurement [36,37]. The most used measurement method was lateral radiograph [35–38] followed by flexicurve ruler [30, 31, 33], kyphometer [36,37], dual inclinometer [34], six-camera motion analysis system [32] and spinal mouse [39]. Among the studies that used radiograph, all measured the Cobb angle. Three studies measured thoracic kyphosis angle, two used the superior endplate of T4 and the inferior endplate of T12 [36,37]. The other study did not indicate the anatomical structure used [35]. One study measured lumbar lordotic angle using the intersection between a line extending from the upper plate of L1 and another extending from the lower plate of L5 [38]. Among the studies that used flexicurve ruler, all measured the angle via the Youdas method. Two measured lumbar lordotic angle marked L1 and S2 and connected this point to each other by a straight line, named L (length). Then, a line was depicted from the deepest point of the curve perpendicular to the L line, named H line (width). Then, a formula was used with the millimetre of the H line and L line [31,33]. Another study measured the kyphosis angle using the same with T2 and T12 as a reference point [30]

### Quality assessment and publication bias

Among the 10 studies, three (30%) scored high quality via the SIGN 50 (++) [32–34], four (40%) scored acceptable quality (+) [30,31,35,39] and three (30%) were low quality (0) [36–38]. The high percentage of poor quality or Not Available (NA) was used for multi-site similarities. Risk of bias was not considered to be serious across studies.

The source population (7/10) [31,33,34,36–39] and the participant recruitment (5/10) [31,36–39] were described.

Among the 10 studies, seven described the staff [31–34,36,37,39], five [31–34 39], and the setting, and all described the intervention characteristics. Most of the studies involved physical therapist, physiotherapist or manual therapists. Treatment locations involved clinics, high schools, senior centres and workplaces (Table 2).

**Table 2:** Quality assessment of included studies.

**SIGN Criteria**	**Poor**	**Adequate**	**Well**	**NA**
Appropriate and clearly focused question	0(0)	0(0)	100(10)	0(0)
Randomization	0(0)	0(0)	100(10)	0(0)
Allocation concealment	50(5)	0(0)	30(3)	20(2)
Blinding	50(5)	0(0)	30(3)	20(2)
Percentage of dropouts	0(0)	0(0)	50(5)	50(5)
Baseline similarities	10(1)	20(2)	70(7)	0(0)
Group differences	10(1)	30(3)	60(6)	0(0)
Outcome reliability /validity	0(0)	0(0)	90(9)	10(1)
Intention to treat	0(0)	0(0)	70(7)	30(3)
Multi-site similarities	30(3)	0(0)	0(0)	70(7)
**EVAT Criteria**	**Poor**	**Adequate**	**Well**	**NA**
Recruitment	30(3)	20(2)	50(5)	0(0)
Participation	30(0)	0(0)	30(3)	40(4)
Model Validity	0(0)	50(5)	50(5)	0(0)

To identify possible publication bias, funnel plots was generated by effect sizes and standard error (S1 Fig. Funnel plot). Egger´s test [47] did not indicate of publication bias (t = 1.447; p = 0.182) and Rosenthal’s fail-safe N [48] shown that 411 additional studies are needed for accumulate a non-significant effect.

### Thoracic kyphosis angle

**Fig 2:**
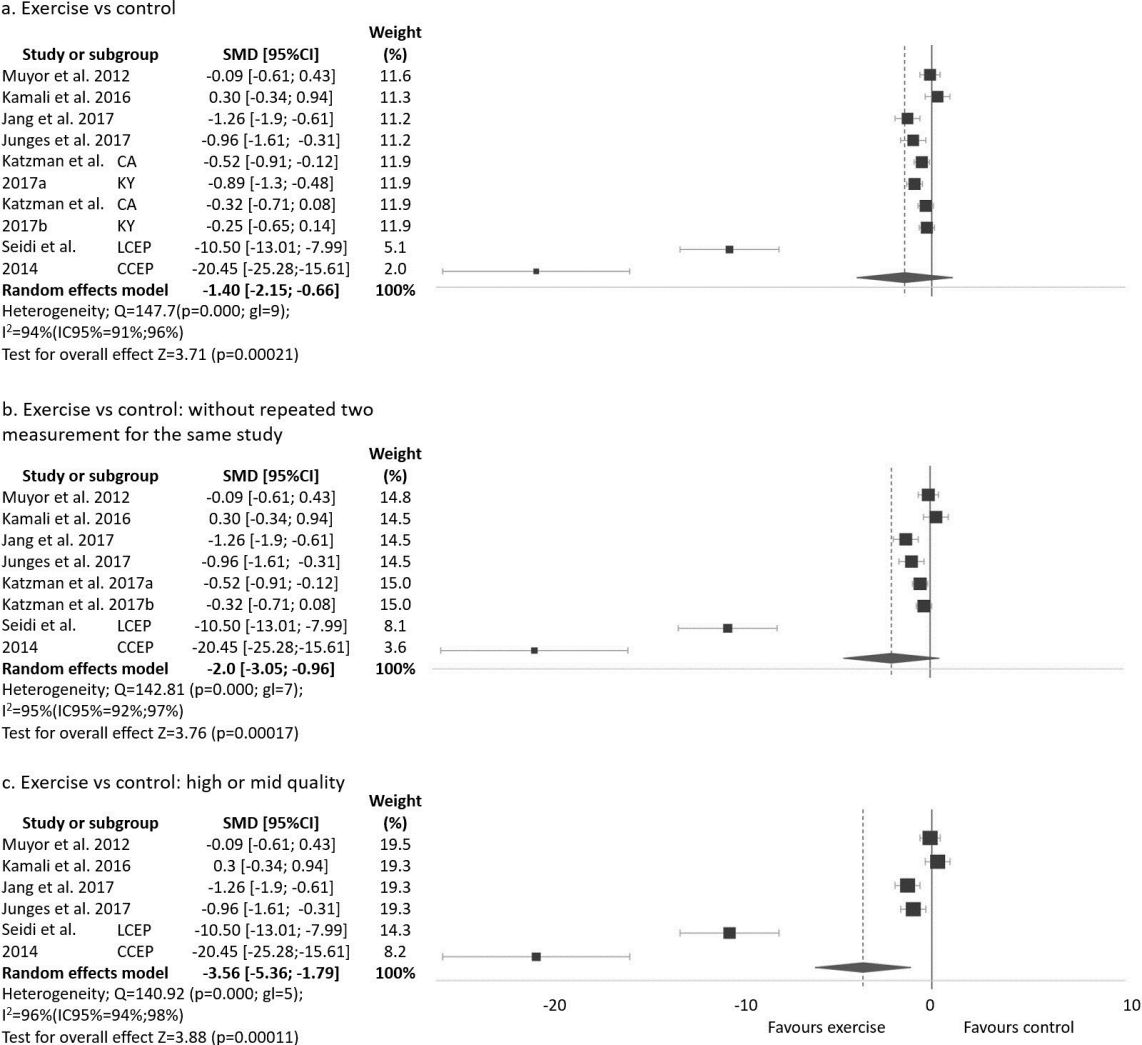
Exercise program versus control group for improvement kyphosis thoracic angle.

Seven studies measured thoracic kyphosis angle [30,32,34–37,39]. Six showed a significant improvement in the angle [30,32 34–37] and one did not show a change [39] after the exercise program. The studies that did not apply any intervention in the CG [30,34–37,39] did not show any change in the CG; however, the study that applied manual therapy in the CG [32] did show an improvement similar to the EG. Two of these studies used two difference measurement methods, Cobb angle and kyphometer [36,37], and others applied two difference exercise programs [30]. Therefore, as a result, 10 groups or subgroups have been analysed and are shown in Fig 2A and in S2 Table.

Thoracic kyphosis angle was assessed in 457 participants (EG = 228; CG = 229). The mean of change between pre- and post-test, standard deviation and number of subjects in both groups (EG and CG) is shown in S2 Table for kyphosis, with SMD (standardized mean difference), 95% confidence interval (95%CI), test for overall effect (z), significance (p) and weight (random). A negative SMD indicated a better evolution of the thoracic kyphosis angle in the EG than the CG. The SMD varied from -20.45 to 0.30. In six of the 10 groups or subgroups of studies, the EG showed better evolution than the CG in thoracic kyphosis angle with statistical significance [30,32,34–36]. The overall SMD ranged from -2.15 to -0.66 with high heterogeneity. Pooled analysis of all interventions demonstrated a large significant improvement in thoracic kyphosis angle in the EG when compared to the CG (SMD = -1.40(95%CI -2.15 a -0.66), p = 0.0002).

Two studies used two measurement methods of the angle [36,37] and they are included in the forest plot. However, the sample and the intervention program are the same and could provide an incorrect result. Therefore, Fig 2B includes just one measurement method of the angle for these studies in order to avoid a possible false result. The Cobb angle measurement was selected because it is the most common method used in the included studies [35–38].

Among seven studies, two presented a low quality score [36,37] as obtained from SIGN 50. Fig 2C presents the analysis without this study and subgroups.

**Fig 3:**
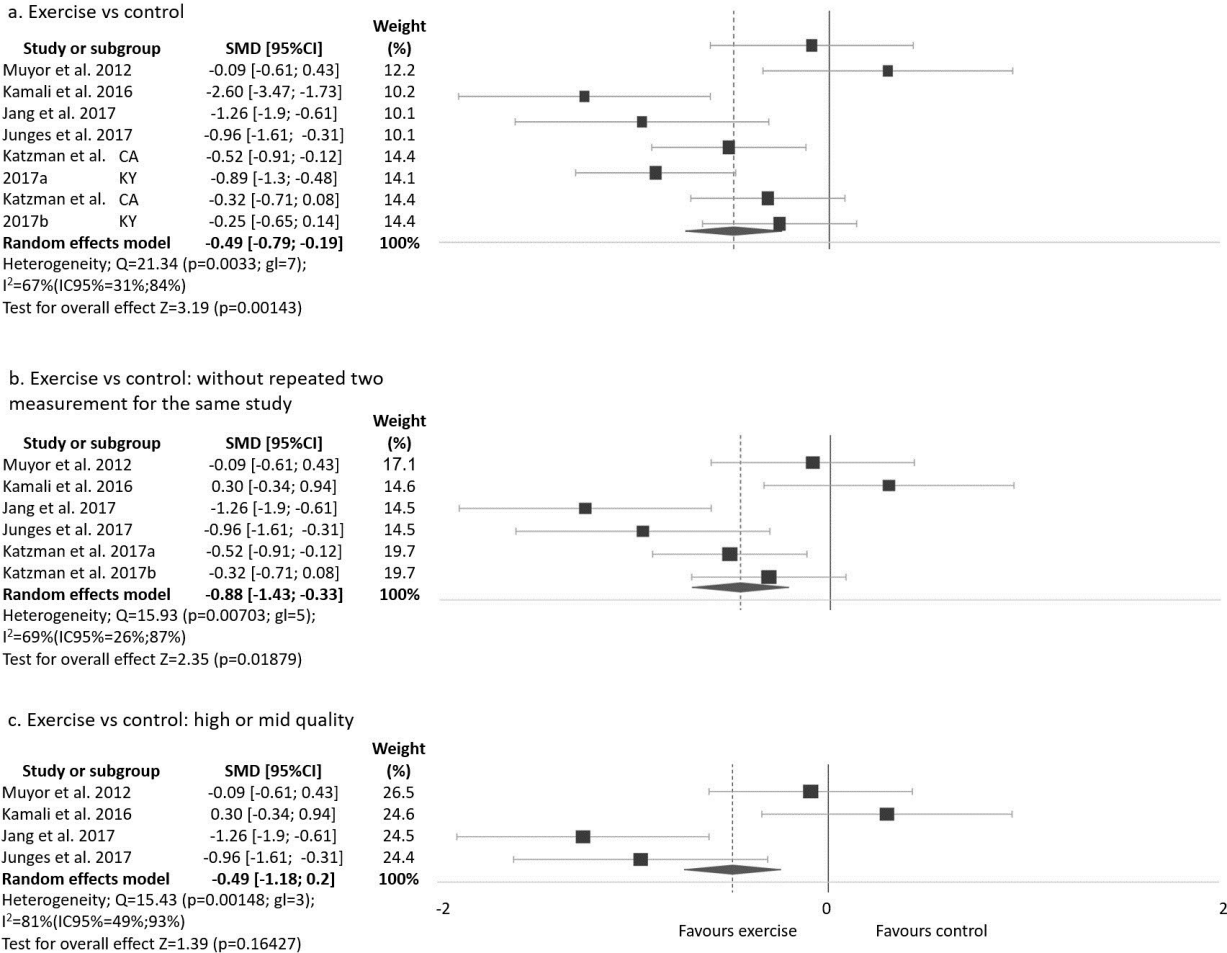
Exercise program versus control group for improvement kyphosis thoracic angle without Seidi et al [30] study.

The same analysis was done removing the two studies or subgroups [30] with the strongest effect on thoracic kyphosis (Fig 3). The analysis of all studies except Seidi et al [30] reduced the heterogeneity and maintained a large significant improvement in thoracic kyphosis angle in the EG when compared to the CG (SMD = -0.49 (95%CI -0.79 a -0.19), p = 0.0014 (Fig 3A). This analysis, without a repeated measurement method, also reduced the heterogeneity and maintained a large significant improvement in thoracic kyphosis angle in the EG when compared to the CG (SMD = -0.46 (95%CI -0.84 a -0.08, p = 0.0187) (Fig 3B). This analysis, without low quality studies, also reduced the heterogeneity and the improvement is no longer significant in the EG when compared to the CG (SMD = -0.49 (95%CI -1.18 a 0.20), p = 0.1642 (Fig 3C).

Analysis was done without the study that applied an alternative program in the CG control group [32]. This analysis showed large significant improvement in thoracic kyphosis angle in the EG when compare to the CG (SMD = -1.65 (95%CI -2.45 a -0.84), p<0.001; I2 = 94%, Chi2 p<0.001). The same analysis without the two studies and subgroups [30] with the strongest effect on thoracic kyphosis, also reduced the heterogeneity and maintained a moderate significant improvement in thoracic kyphosis angle in the EG when compared to the CG (SMD = -0.57 (95%CI -0.85 a -0.29), p<0.001, I2 = 61%, Chi2 p = 0.0181). On the other hand, the analysis without the study that applied an alternative program in the CG [32] and just included high or moderate quality obtained from a SIGN 50 score [30, 34, 35, 39] showed a large significant improvement in this measure (thoracic kyphosis angle) in the EG versus CG although the heterogeneity is high (SMD = -4.77 (95%CI -7.00 a -2.54), p<0.001, I2 = 93%, Chi2 p<0.001).

### Lumbar lordotic angle

**Fig 4:**
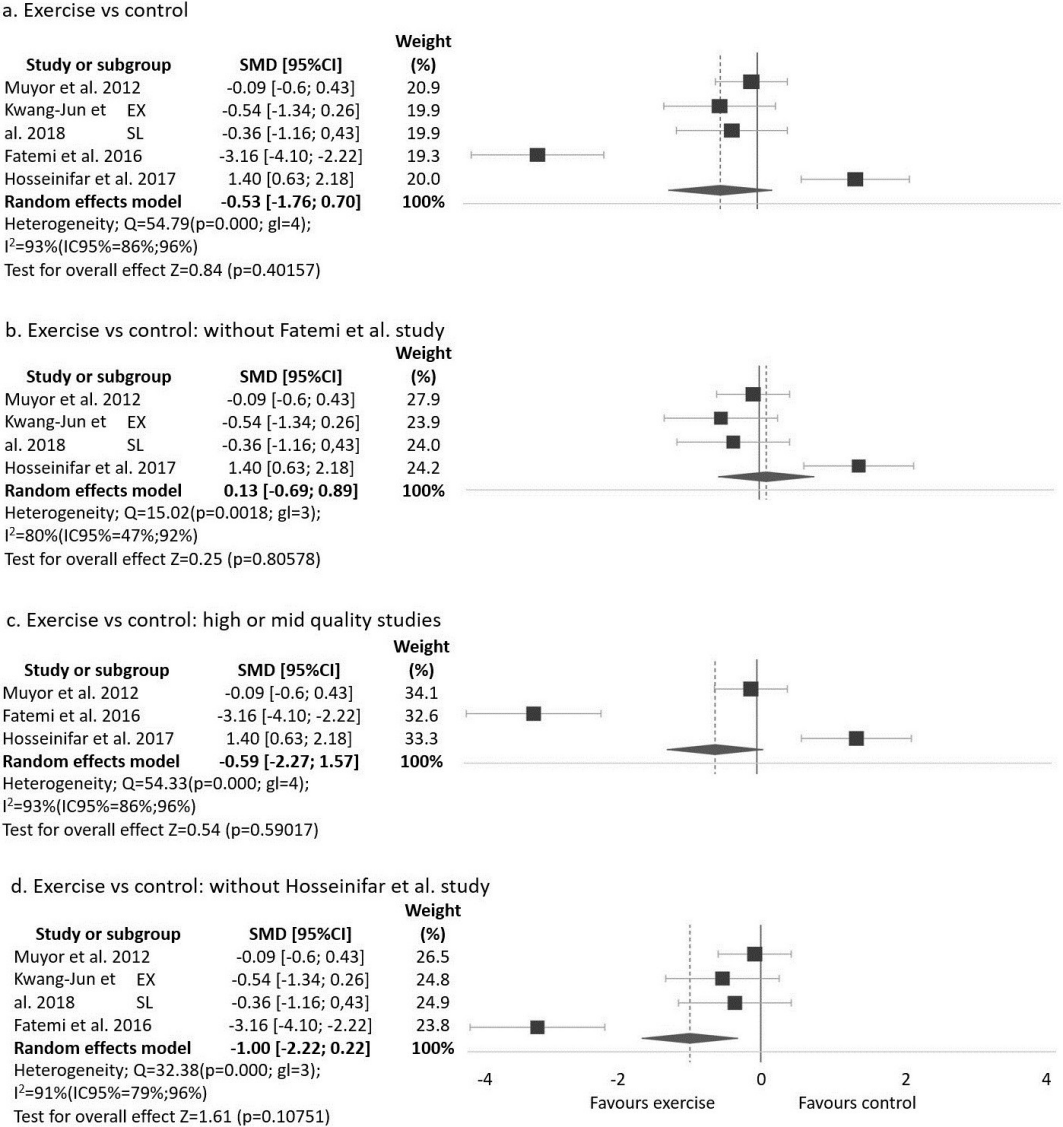
Exercise program versus control group for improvement lordosis lumbar angle.

Four studies assessed lumbar lordotic angle [31,33,38,39]. Two of the four studies that assessed this angle showed significant improvement in the angle after the exercise program [31,33] and the other two studies did not show change in the angle [38,39]. Three studies did not apply the intervention in the CG and one applied a physiotherapy protocol [33] showing a similar improvement to the exercise program of the EG. One of these studies applied two different exercise programs and then the authors compared results with a CG [38]. The other three studies just applied one exercise program. Therefore, as a result, five groups or subgroups were analysed and are shown in Fig 4A and S3 Table.

Lumbar lordotic angle was assessed in 159 participants (EG = 83; CG = 76). Additional analysis info is shown in the S3 Table. The SMD varied from -3.16 to 1.40. In one of the five groups or subgroups of studies, the EG showed better evolution than CG in lumbar lordotic angle with statistical significance [31]. One study, which compared an exercise program with a physiotherapy program, showed improvement after applying the two programs; however, the improvement was higher in the physiotherapy program than in the exercise program [33].

Pooled analysis of all interventions show moderate improvement in lumbar lordotic angle in the EG when compared to the CG, although not significantly (SMD = -0.53 (95% CI -1.76 a -0.70), p = 0.4015).

The removal of one study [31] with the strongest effect on lumbar lordosis angle, reduced the heterogeneity and the improvement in (SMD = 0.10 (95%CI -0.69 a 0.89), p = 0.8057, I2 = 80%, Chi2 p = 0.0018) (Fig 4B).

Among the four studies that assessed lumbar angle, one of them presented a low quality [38] score from SIGN 50. Fig 4C presents the analysis without this study and subgroups.

At for the thoracic kyphosis curve, analysis was done without the study that applied an alternative program in the CG [33] showing large no significant improvement in lumbar lordotic angle in the EG when compare to the CG (SMD = -1 (95% CI -2.22 a 0.22), p = 0.1075) (Fig 4D).

## Discussion

The purpose of this meta-analysis was to determine the effect of exercise on thoracic kyphosis and lumbar lordotic angle. There was a large, statistically significant effect of exercise improving thoracic kyphosis angle, although there was no significant effect of exercise on lumbar lordotic angle.

This is the first systematic review and meta-analysis that analyses the effect of the exercise on these angles. Only one systematic review evaluated previous research to determine if exercise improves hyperkyphosis by decreasing the angle of thoracic kyphosis in adults aged above 44 years [53]. These authors included 13 studies in their systematic review showing in eight of them improvement in the angle. We included fewer studies because our inclusion criteria selected only RCTs and exercise interventions involving strengthening, stretching, endurance or/and resistance. Bansal et al [53] included all types of study and all types of exercise interventions (active exercise and passive mobilization by a physical therapist). Five of the included studies were pre-post intervention designs and one was a follow-up study. Four of these six studies showed significant improvement in kyphosis angle after the intervention and another showed a 5% improvement, but did not report whether this change was significant.

Most of the studies were carried out with women [31,32,34,35,38,39]. In two studies more women participated than men [36, 37]. In another participation was the same [30], and the last did not identify the sex [33]. Bansal et al [53] reported a similar majority participation by woman in the studies included in their review. This aspect should be considered because the prevalence of hyperkyphosis or hyperlordosis for women or men could influence results. Some studies have reported a difference between sexes in kyphosis and lordosis angle, showing a higher angle of kyphosis in males and a higher angle of lordosis in females [54,55]. However, others have found differences just in kyphosis [56] or just in lordosis [57]. Only one study researched the effect of the exercise on kyphosis angle differences between sexes, finding the same result in both sexes [36].

In connection with the inclusion and exclusion criteria, all of the studies that evaluated thoracic kyphosis angle, except one, showed hyperkyphosis as an inclusion criteria, defining hyperkyphosis as a thoracic angle as more than 45° [32,35] more than 42° [30] or more than 40° [34,36,37]. Bansal et al also included hyperkyphosis as an inclusion criteria, defining it as an angle equal to or above 40°. In this sense, some authors considered hyperkyphosis a thoracic curve equal or above 40° [53,58], while other authors indicated it as above 45° [59–61] and others above 50° [62]. On the other hand, just one of the four studies that evaluated lumbar lordotic angle reported as an inclusion criteria evidence of lumbar hyperlordosis [31], but did not define it. Authors reported lumbar hyperlordosis as an angle above 40° [60, 61, 63] or above 45° [64].

All studies used stretching and/or strengthening exercises in their program as the main intervention. Regarding thoracic kyphosis angles, this meta-analysis showed a large significant improvement after exercise programs. Just one of the studies did not show significant change [39] after the exercise program. This study [39] is the only one that applied only a stretching program without strengthening. The other studies included strengthening exercise in their program. Hyperkyphosis has been associated with low values of hamstring flexibility [22–24] and a lack of abdominal and paravertebral strengthening [24–26]. However, this meta-analysis suggests that strengthening could be more relevant than stretching for the thoracic curve, or at least it is necessary to work both to reduce the curve of the thoracic angle. More research is necessary.

In connection with lumbar lordosis, the meta-analysis showed moderate but not significant improvement in lumbar lordotic angle in the EG. Although the analysis did not show a significant effect of the exercise on lordosis, one included study did show [31] and another study [33] which combined an exercise program with a physiotherapy program showed improvement after applying the two programs; however, the improvement was higher in the physiotherapy program than in the exercise program. On the other hand, these two studies [31,33] applied stretching and strengthening exercise in their intervention. By contrast, the studies that did not show improvement only applied stretching [39] or strengthening exercise [38] in their program. In this regards, hyperlordosis is related to a shortening of psoas iliac (flexor of hip) [24,27,28] and a lack of abdominal and paravertebral strengthening [24,27] and hyperlordosis is associated with a short hamstring muscle [29]. Consistent with Karimi and Rahnama [65] we conclude that both stretching and strengthening are relevant in lumbar lordosis.

Kamali et al [32] and Hosseinifar et al [33] applied stretching and strengthening exercise programs. These studies applied an alternative program of manual therapy [32] and a physiotherapy protocol [33] in the CG, and showed similar significant results. This suggests that exercise programs that include stretching and strengthening exercise could have similar effects in hyperkyphosis and hyperlordosis as manual therapy and physiotherapy protocol. However, more RCTs comparing these programs are necessary.

The present review showed that for the EG the mean duration of the exercise program was 12.5 weeks (2–30 weeks), and mean frequency was 3 sessions per week (range 2–6). The duration of the program in the review by Bansal et al [53] was from 8 to 12 weeks and the frequency from 2 to 3 days per week. Hosseinifar et al [33] applied 6 sessions per week during 2 weeks and Katzman et al [37] applied three sessions per week during 6 months, finding significant improvement in both studies and with similar changes in the angle. The study that found more improvement in the thoracic angle applied an intervention of 2 days/week for 12 weeks [30] and the study that found the same in the lumbar angle applied an intervention 3 days/week for 8 weeks [31]. As discussed previously, strengthening of muscle could be more relevant than stretching for hyperkyphosis, and equally relevant to hyperlordosis; however this could be dependent on changing the frequency per week in order to obtain change in each angle.

Bansal et al [53] showed in their review more change in the kyphosis angle after a program of 3 days per week; however, they indicated that these finding were found in two articles that lack a minimum quality to establish conclusions. This makes it necessary to research the frequency and minimum duration of the exercise program to find change in the sagittal spinal curve.

Among the 10 studies included, three showed low quality [36–38] obtained from SIGN 50 score. When these studies are removed from the pooled analysis, the significant improvement rises in the thoracic kyphosis angle and a maintained moderate but not significant improvement in lordosis. Bansal et al [53] concluded that the positive effect observed in the high-quality studies suggest that exercise shows some benefit in the thoracic kyphosis angle and supported the need for more developed RCTs. We support this conclusion and add that there is a similar need in connection with lumbar lordosis.

Thoracic and lumbar angles are measured with a radiologic image and the gold standard Cobb´s angle [66]. However, this method requires X-ray radiation [65,66] has limited portability [67, 68] and is expensive [69, 70]. Likewise, alternative methods have been used, [71, 72] as in this meta-analysis, such as: flexicurve ruler [30,31,33], kyphometer [36,37], dual inclinometer [34], six-camera motion analysis system [32] and spinal mouse [39]. The validity and reliability of these methods for measuring back angles have been assessed. Dual inclinometer has shown high validity (ICC = 0.81 [71]) and reliability in measurement of kyphosis angle (ICC = 0.97 [73], ICC = 0.87–0.92[74]) and lordosis angle (ICC = 0.96[75], ICC = 0.95[76]). This method shows some advantage, such as automated calculation, to lower measurement error and increase efficiency [77]. Flexicurve ruler is a simple inexpensive tool, and permits a quick clinical measurement of the sagittal plane of back angle [72]. This method has shown a moderate-high validity (ICC = 0.96 [78], kyphosis: r = 0.72, lordosis: r = 0.60[69]) and high reliability (ICC = 0.891–0.967[79], kyphosis: ICC = 0.820–0.942; lordosis: ICC = 0.783–0.831[69]).

Kyphometer is a non-invasive method, clinically feasible in cost and ease of application [79]. There is moderate validity for this method (ICC = 0.622–0.762[80], ICC = 0.759[81], ICC = 0.622[80]) and high reliability (ICC = 0.98[80], ICC = 0.91–0.94[82], ICC = 0.95–0.97[82], ICC = 0.84–0.92[81], ICC = 0.98[80] ICC = 0.992–0.890[79]). Six-camera motion analysis system or photogrammetry is a non-invasive method to measure spinal curve with the advantage of being able to be used to evaluate spinal curvature in movement [83]. It shows high validity (kyphosis: ICC = 0.967–0.975; lordosis: ICC = 0.900–0.912[83], r = 0.76[84]) and high reliability (kyphosis: ICC = 0.988; lordosis: ICC = 0.985[83], thoracic: ICC = 0.93–0.97; lumbar: ICC = 0.85–0.90[85]). Spinal mouse is a non-invasive device that provides a short time to assessment, is low cost and safe [86]. It has been demonstrated to have moderate-high validity (0.865–0.989 and 0.765–0.991[86], thoracic kyphosis: r = 0.81, lumbar lordosis: r = 0.86[87], lumbar lordosis: ICC = 0.872–0.962[88]) and moderate-high reliability (kyphosis: ICC = 0.73–0.88; lordosis: ICC = 87–93[89]; kyphosis: ICC = 81.87; lordosis: ICC = 84–94[90]).

The main point of the current study was to analyse the effects of exercise on thoracic kyphosis angle and lumbar lordotic angle. To our knowledge, there are no meta-analyses that analyse the effects of the exercise program on sagittal spinal curvatures and the published systematic review only assessed the thoracic kyphosis angle in older patients. The first statistical analysis of the present meta-analysis used all the measurement and method used in each study. In addition, in order to avoid false result, the present meta-analysis performed more additional statistics analysis, removing some study. It was performed an analyses using just one measurements or method of each study. It was performed other analysis using only high or mid quality studies. In order to compare just the exercise program groups with control groups, another analysis without the studies that applied an alternative therapy in the control group was performed. Moreover, analysis without the studies that showed the strongest effect on the angles. Other strengths of this review are the rigorous process of the review, the inclusion and exclusion criteria and selection of only RCTs. These aspects provide strong confidence it the main findings.

These study results should be considered in light of certain limitations. Although the results shown in most of the studies are similar, and publication bias was not identified most of the analyses showed moderate to high heterogeneity. This could be because the formulas used to calculate the standard deviation of the change of the angle when not provided in the article provides conservative results and reports of a high standard deviation, which could influence heterogeneity. In addition, the small number of subjects in some analyses could explain the high heterogeneity [91]. In addition, the heterogeneity could be due to the different intervention in the experimental group and the different treatment in control group. Some studies compare an exercise program group with a control group; and other studies compare exercise program group with other type of therapy group [92].

## Conclusion

This meta-analysis found a large, statistically significant, effect of exercise improving thoracic kyphosis angle and no significant effect on lumbar lordotic lumbar angle. On one hand, that suggests that strengthening could be more relevant than the stretching for the thoracic curve, or at least it is necessary to work both to reduce the curve of the thoracic angle. In addition, this studies suggests that stretching and strengthening are relevant in the lumbar lordotic angle. This systematic review suggests a frequency of 2–3 session per week during 8–12 weeks in order to find improvement in the sagittal spinal curvatures. It is necessary to conduct more RCTs that assess the effect of strengthening and/or stretching program on thoracic kyphosis and lumbar lordotic angle in order to establish the type of the exercise that it is best for maintaining the sagittal disposition inside normal ranges, and to compare different frequencies and durations of the exercise program.

## Supporting information

S1 Fig Funnel plot. (TIF)

S1 Table Search term applied. (DOCX)

S2 Table Mean of change pre-post-test of kyphosis thoracic angle and standard deviations of EG and CG in the included studies. N = number of subject; M = mean; SD = standard deviation; SMD = standardized mean difference; 95%CI = confidence interval, z = test for overall effect; p = significance; W = weight.
(DOCX)

S3 Table Mean of change pre-post-test of lordosis lumbar angle and standard deviations of EG and CG in the included studies. N = number of subject; M = mean; SD = standard deviation; SMD = standardized mean difference; 95%CI = confidence interval, z = test for overall effect; p = significance; W = weight.
(DOCX)

S1 File PRISMA checklist. (DOC)
