# Supplementary figures and images for: Effects of exercise programs on kyphosis and lordosis angle: A systematic review and meta-analysis
Source: PLoS One. 2019 Apr 29;14(4):e0216180. doi: 10.1371/journal.pone.0216180 (PMC6488071; doi:10.1371/journal.pone.0216180)

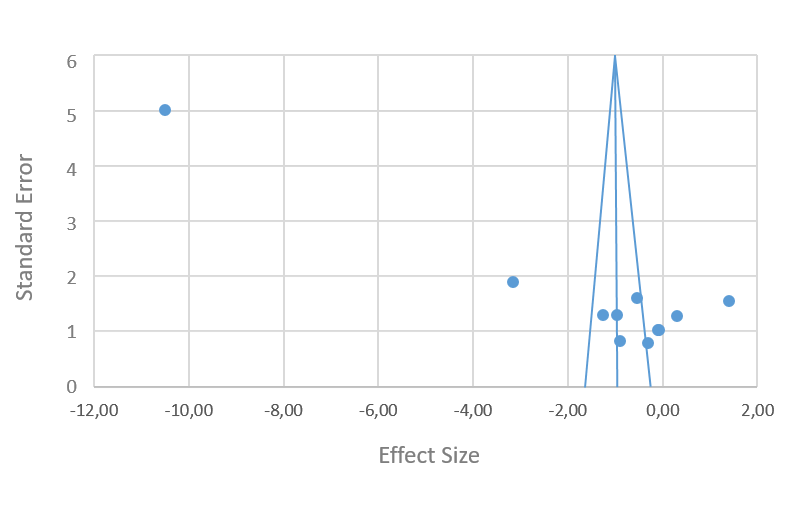

Supplement: S1 Fig — (TIF) [file pone.0216180.s001.tif]
