# Supplementary material for: Effects of exercise programs on kyphosis and lordosis angle: A systematic review and meta-analysis
Source: PLoS One. 2019 Apr 29;14(4):e0216180. doi: 10.1371/journal.pone.0216180 (PMC6488071; doi:10.1371/journal.pone.0216180)
Supplement: S1 Table — (DOCX) [file pone.0216180.s002.docx]

| **S1 Table. Search term applied** | |
| --- | --- |
| Database | Search terms and MeSH terms |
| Pub med  Search restrictions:   - All text - Clinical trial | (lordosis and exercise) OR (lordosis and physical activity) OR (lordosis and program*) OR (lordosis and training) OR (kyphosis and exercise) OR (kyphosis and physical activity) OR (kyphosis and program*) OR (kyphosis and training) OR (spinal curv* and physical activity) OR (spinal curv* and exercise) OR (spinal curv* and training) (spinal curv* and program) OR (sagittal spinal and exercise) OR (sagittal spinal and physical activity) OR (sagittal spinal and training) |
| Cochrane Search restrictions:   - All text | (lordosis and exercise) OR (lordosis and physical activity) OR (lordosis and program*) OR (lordosis and training) OR (kyphosis and exercise) OR (kyphosis and physical activity) OR (kyphosis and program*) OR (kyphosis and training) OR (spinal curv* and physical activity) OR (spinal curv* and exercise) OR (spinal curv* and training) (spinal curv* and program) OR (sagittal spinal and exercise) OR (sagittal spinal and physical activity) OR (sagittal spinal and training) OR (sagittal spinal and program*) |
| WOS | TI=((lordosis AND (exercise OR pyhysical activity OR program* OR training)) OR (kyphosisAND (exercise OR pyhysical activity OR program* OR training)) OR (spinalcurv* AND (exercise OR pyhysical activity OR program* OR training)) OR (sagittal spinal AND (exercise OR pyhysical activity OR program* OR training))) |
| EBSCO | (lordosis and exercise) OR (lordosis and physical activity) OR (lordosis and program*) OR (lordosis and training) OR (kyphosis and exercise) OR (kyphosis and physical activity) OR (kyphosis and program*) OR (kyphosis and training) OR (spinal curv* and physical activity) OR (spinal curv* and exercise) OR (spinal curv* and training) (spinal curv* and program) OR (sagittal spinal and exercise) OR (sagittal spinal and physical activity) OR (sagittal spinal and training) OR (sagittal spinal and program*) |
| PEDro |  |
| Search 1 | Abstract & Title: spinal curv*; therapy: fitness training; body part: thoracic spine; method: clinical trial |
| Search 2 | Abstract & Title: spinal curv*; therapy: fitness training; body part: lumbar spine, sacro-iliac joint or pelvis; method: clinical trial |
| Search 3 | Abstract & Title: sagittal spinal; therapy: fitness training; body part: thoracic spine; method: clinical trial |
| Search 4 | Abstract & Title: sagittal spinal; therapy: fitness training; body part: lumbar spine, sacro-iliac joint or pelvis; method: clinical trial |
| Search 5 | Abstract & Title: spinal curv*; therapy: strength training; body part: thoracic spine; method: clinical trial |
| Search 6 | Abstract & Title: spinal curv*; therapy: strength training; body part: lumbar spine, sacro-iliac joint or pelvis ; method: clinical trial |
| Search 7 | Abstract & Title: sagittal spinal; therapy: strength training; body part: thoracic spine; method: clinical trial |
| Search 8 | Abstract & Title: sagittal spinal; therapy: strength training; body part: lumbar spine, sacro-iliac joint or pelvis; method: clinical trial |
| Search 9 | Abstract & Title: spinal curv*; therapy: stretching, mobilisation, manipulation, massage; body part: thoracic spine; method: clinical trial |
| Search 10 | Abstract & Title: spinal curv*; therapy: stretching, mobilisation, manipulation, massage; body part: lumbar spine, sacro-iliac joint or pelvis ; method: clinical trial |
| Search 11 | Abstract & Title: sagittal spinal; therapy: stretching, mobilisation, manipulation, massage; body part: thoracic spine; method: clinical trial |
| Search 12 | Abstract & Title: sagittal spinal; therapy: stretching, mobilisation, manipulation, massage; body part: lumbar spine, sacro-iliac joint or pelvis; method: clinical trial |
