# Supplementary material for: Effects of exercise programs on kyphosis and lordosis angle: A systematic review and meta-analysis
Source: PLoS One. 2019 Apr 29;14(4):e0216180. doi: 10.1371/journal.pone.0216180 (PMC6488071; doi:10.1371/journal.pone.0216180)
Supplement: S2 Table — N = number of subject; M = mean; SD = standard deviation; SMD = standardized mean difference; 95%CI = confidence interval, z = test for overall effect; p = significance; W = weight. (DOCX) [file pone.0216180.s003.docx]

| **S2 Table. Mean of change pre-post-test of kyphosis thoracic angle and standard deviations of EG and CG in the included studies** | | | | | | | | | | | |
| --- | --- | --- | --- | --- | --- | --- | --- | --- | --- | --- | --- |
|  | **Experimental Group** | | | **Control Group** | | | **SMD** | **95%CI** | **z** | **p** | **W (%)** |
| **Study or subgroup of study** | **N** | **M** | **SD** | **N** | **M** | **SD** |  |  |  |  |  |
| Muyor et al 2012[39] | 27 | 0.66 | 7.72 | 31 | 1.28 | 6.06 | -0.09 | -0.61; -0.09 | -0.61 | 0.732 | 11.6 |
| Kamali et al 2016[32] | 16 | 2.51 | 1.92 | 23 | 3.17 | 2.35 | 0.30 | -0.34; 0.94 | 0.92 | 0.356 | 11.3 |
| Jang et al 2017[34] | 22 | -2.20 | 0.74 | 22 | 0.08 | 2.46 | -1.26 | -1.9; -0.61 | 3.79 | 0.000 | 11.2 |
| Junges et al 2017[35] | 22 | -8.00 | 7.50 | 19 | -0.61 | 7.90 | -0.96 | -1.61; -0.31 | 2.90 | 0.003 | 11.2 |
| Katzman et al 2017[36] |  |  |  |  |  |  |  |  |  |  |  |
| Cobb angle | 53 | -1.40 | 3.71 | 48 | 0.30 | 2.74 | -0.52 | -0.91; -0.12 | 2.55 | 0.010 | 11.9 |
| Kyphometer | 53 | -3.80 | 5.57 | 48 | 1.00 | 5.13 | -0.89 | -1.3; -0.48 | 4.28 | 0.000 | 11.9 |
| Katzman et al 2017[37] |  |  |  |  |  |  |  |  |  |  |  |
| Cobb angle | 51 | -3.30 | 12.02 | 48 | -0.30 | 5.48 | -0.32 | -0.71; 0.08 | 1.57 | 0.116 | 11.9 |
| Kyphometer | 51 | -3.80 | 14.02 | 48 | -0.90 | 7.72 | -0.25 | -0.65; 0.14 | 1.26 | 0.208 | 11.9 |
| Seidi et al 2014[30] |  |  |  |  |  |  |  |  |  |  |  |
| EG1: LCEP | 19 | -5.04 | 0.44 | 19 | 0.62 | 0.62 | -10.50 | -13.01; -7.99 | 8.21 | 0.000 | 5.1 |
| EG2: CCEP | 18 | -12.25 | 0.51 |  |  |  | -20.45 | -25.28;-15.61 | 8.29 | 0.000 | 2.0 |
| Legend: N= number of subject; M=mean; SD=standard deviation; SMD=standardized mean difference; 95%CI=confidence interval, z=test for overall effect; p=significance; W=weight | | | | | | | | | | | |
