# Supplementary material for: Effects of exercise programs on kyphosis and lordosis angle: A systematic review and meta-analysis
Source: PLoS One. 2019 Apr 29;14(4):e0216180. doi: 10.1371/journal.pone.0216180 (PMC6488071; doi:10.1371/journal.pone.0216180)
Supplement: S3 Table — N = number of subject; M = mean; SD = standard deviation; SMD = standardized mean difference; 95%CI = confidence interval, z = test for overall effect; p = significance; W = weight. (DOCX) [file pone.0216180.s004.docx]

| **S3 Table. Mean of change pre-post-test of lordosis lumbar angle and standard deviations of EG and CG in the included studies** | | | | | | | | | | | |
| --- | --- | --- | --- | --- | --- | --- | --- | --- | --- | --- | --- |
|  | **Experimental Group** | | | **Control Group** | | | **SMD** | **95%CI** | **z** | **p** | **W(%)** |
| **Study or subgroup of study** | **N** | **M** | **SD** | **N** | **M** | **SD** |  |  |  |  |  |
| Muyor et al 2012[39] | 27 | 0.80 | 4.35 | 31 | 1.17 | 4.19 | -0.09 | -0.60; 0.43 | 0.33 | 0.741 | 20.9 |
| Kwang-Jun et al 2018[38] |  |  |  |  |  |  |  |  |  |  |  |
| Exercise | 20 | -0.30 | 0.49 | 9 | 0.10 | 1.13 | -0.54 | -1.34; 0.26 | 1.32 | 0.185 | 19.9 |
| Sling | 20 | -0.20 | 0.66 |  |  |  | -0.36 | -1.16; 0.43 | 0.90 | 0.370 | 19.9 |
| Fatemi et al 2015[31] | 20 | -6.08 | 1.66 | 20 | -0.08 | 2.11 | -3.16 | -4.10; -2.22 | 6.57 | 0.000 | 19.3 |
| Hosseinifar et al 2017[33] | 16 | 3.64 | 1.11 | 16 | 1.64 | 1.68 | 1.40 | 0.63; 2.18 | 3.54 | 0.000 | 20.0 |
| Legend: N= number of subject; M=mean; SD=standard deviation; SMD=standardized mean difference; 95%CI=confidence interval, z=test for overall effect; p=significance; W=weight | | | | | | | | | | | |
